# Sensing Bioavailable Water Content of Granulated Matrices: A Combined Experimental and Computational Study

**DOI:** 10.3390/bios13020185

**Published:** 2023-01-25

**Authors:** Ria Ghosh, Neha Bhattacharyya, Amrita Banerjee, Lopamudra Roy, Debdatta Mukherjee, Soumendra Singh, Arpita Chattopadhyay, Tapan Adhikari, Samir Kumar Pal

**Affiliations:** 1 Department of Chemical, Biological and Macromolecular Sciences, S. N. Bose National Centre for Basic Sciences, Kolkata 700106, India; 2 Department of Radio Physics and Electronics, University of Calcutta, Kolkata 700009, India; 3 Department of Physics, Jadavpur University, Kolkata 700032, India; 4 Department of Applied Optics and Photonics, University of Calcutta, Kolkata 700009, India; 5 Department of Biochemistry and Biophysics, University of Kalyani, Kalyani 741235, India; 6 Department of Basic Science and Humanities, Techno International, Kolkata 700156, India; 7 Indian Institute of Soil Science Nabibagh, Bhopal 462038, India

**Keywords:** non-invasive, non-contact, soil moisture, relative humidity, water potential, nano sensor

## Abstract

This paper represents the synthesis, characterization and validation of a cobalt chloride functionalised nano-porous cellulose membrane, a unique sensor for non-contact measurement of water potential in various biomedical and environmentally important matrices. The developed nano sensor, along with associated electronic components, is assembled as a prototype device called “MEGH” (Measuring Essential Good Hydration) to measure essential hydration of matrices of both environmental and biomedical importance, including soil and human skin. The relative humidity above the soil surface in equilibrium with the soil moisture has been studied for both hydrophobic and hydrophilic soil types. Our studies confirm that the percentage of water available to plants is greater in hydrophobic soil rather than in hydrophilic soil, which has also been corroborated using simulation studies. Furthermore, the requirement of hydration in human skin has also been evaluated by measuring the water potential of both dry and moist skin.

## 1. Introduction

Timed delivery of hydration from a water-retaining matrix, triggered by the requirement, is a unique, and novel idea. Potential applications include but are not limited to the synthesis of hydrogels in biomedical use, control of micro-irrigation by reducing wastage of water [1], and modern greenery-reclaiming technology in desert cities through the measurement and management of moisture. Therefore, authentic estimation of soil moisture level is crucial for maintaining a sustainable global environment.

In recent times, indirect techniques for soil moisture measurement have become increasingly convenient as alternatives to the conventional time-consuming, elaborate, expensive, direct methods such as the in situ thermogravimetric method and electromagnetic (EM) sensor networks [1,2]. Although the gravimetric method is a reliable and accurate technique, it may face unpredictability due to variation in soil quality, sudden loss in mechanical contact between sensors, or lack of proper calibration technique [1]. Amongst the indirect techniques, time domain reflectometry and frequency domain reflectometry are reported to be the most accurate methods [3]. However, these methods suffer from low spatial representation and can be further improved by using cosmic-ray neutron sensing and global positioning systems such as emerging technologies [3]. Nonetheless, all the above-mentioned techniques lack popularity, especially in farming communities because of their high cost and complex operating protocols. A comprehensive list of available water potential-sensing devices is displayed in Table 1.

In our study, an indirect, non-contact pathway was explored where relative humidity (RH) or the water potential (WP) in soil was used as a marker to find out the moisture level in the matrix. In proximity to soil, RH and WP values stay in equilibrium with soil moisture; hence, they are used as essential parameters to estimate the amount of moisture content in a soil matrix (host of water) [10,11]. Measurement of water potential (*φ*), which is primarily the indirect estimation of the amount of water present in capillary and absorptive surfaces within the solid matrix, is more convenient than time-consuming gravimetric and volumetric techniques [1]. The water potential measurement is also the basis of several non-invasive hydration measurements, including psychrometry [12]. Recently, significant advancements in the direction of design and performance of psychrometric water potential measurement have been researched [13], where psychrometers are used to measure WP (*φ*) following the well-known physical–chemical foundation of Kelvin’s work [14]. At present, psychrometric sensors are found to be a good solution, as they measure the humidity of air that remains in equilibrium with a sample of material-containing water molecules and respond to the total water potential. In Kelvin’s Equation, the relation between WP and RH are theoretically expressed as [1,14].

(1) $$\varphi =\frac{RT}{V_{w}}\ln\frac{e}{e_{0}}$$

where the water potential (*φ*) in Pa is related to *R*, the universal gas constant (8.314 J mol K^−1^), *e*/*e*_0_; the relative humidity is expressed as a fraction, *T* is the absolute temperature, and *V_w_* is the molar volume of water (1.8 × 10^−5^ m^3^ mol^−1^).

In this context, it is worth mentioning the significance of controlled hydration in the human body [15], where water comprises 75% body weight in infants to 55% in the elderly and is essential for cellular homeostasis. The ability to measure hydration in a biomedical application in a non-invasive manner would lead to numerous clinical advantages. Notably, the devices based on Terahertz Time-Domain-Spectroscopy (THz-TDS) systems have the potential to measure the hydration in biological tissues and chemical analysis [16,17,18,19,20]. However, THz radiation gets absorbed by water itself and consequently does not penetrate moist tissue to any significant depth [17,21]. To circumvent these challenges, a novel approach was adopted in our study, where cobalt chloride (CoCl_2_) functionalised micro membrane (FMM) (porous cellulose membrane) was synthesised and characterized to measure the non-contact water potential of various matrices of biomedical and environmental importance. To ensure the multipurpose application of the developed sensor, it was tested across the matrix environments, such as in a cotton pad and in various kind of soils. For the cotton pad, overall hydration of human body was measured by sensing WP of the cotton pad in contact with human skin. For soils, the moisture-retaining capability and amount of plant-available water in various types of soil matrices (sand, loam) was investigated. It has to be noted that several biomedical components, including cotton pads for wound dressing, and several applications for maintaining hygiene need water retention capability. Our developed device is also intended to use for the sensing of hydration retention in the system.

## 2. Materials and Methods

### 2.1. Chemicals

Cobalt chloride, formic acid, and dioxane was obtained from Sigma-Aldrich, St. Louis, MO, USA. All reagents were used without further purification. Analytical-grade chemicals were used and all the solutions were prepared in Millipore water.

### 2.2. Preparation of Sensor for Moisture Detection

**Figure 1:**
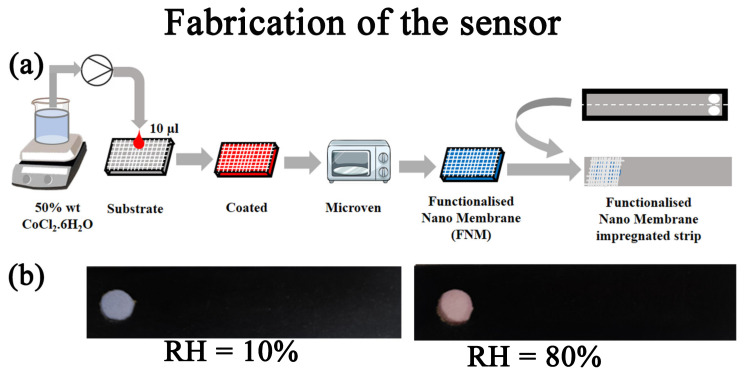
Design of the nano sensor: (**a**) Schematic representation of synthesis of the functionalised nano membrane. (**b**) Indigenously developed strips exposed to a humidity condition on 10% and 80% in a controlled humidity chamber.

An amount of 50 wt% CoCl_2_·6H_2_O (Sigma, St. Louis, MO, USA) was dissolved in water and drop-casted in a cellulose membrane of 205 µm thickness with pore size 20–25 µm. The cellulose membrane was kept in a microven for 10 min to obtain the Functionalised Micro Membrane (FMM). (Figure 1a). Figure 1b depicts the colour change in FMM, where with increase in moisture or RH, the sensor turns to red from blue.

### 2.3. Fabrication and Design of the Paper-Based Sensor Strips (Patent Pending)

**Figure 2:**
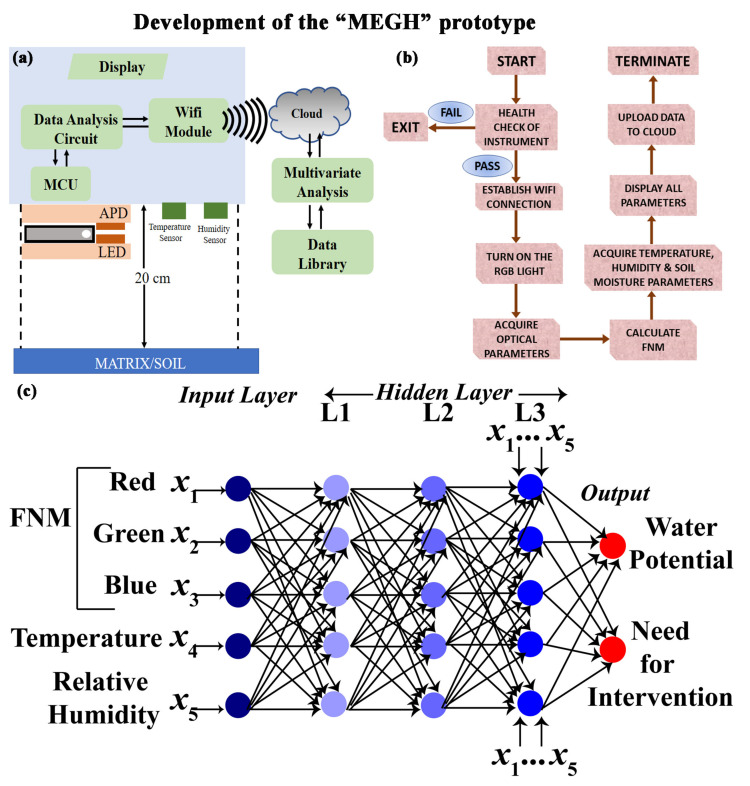
Working principle of MEGH device: (**a**) Schematic representation of the MEGH device along with its components. (**b**) Workflow chart for the operation of the device. (**c**) Artificial neural network incorporated into the software of the device for determination of water potential and requirement of intervention.

Filter papers with dimension 1 cm^2^ were used as the base of the sensor strip. A pre-defined hydrophilicity/porosity index of the filter papers was maintained throughout the experiments (pore size: 20–25 μm, thickness: 205 μm, ash: ≤0.06%, and basic weight: 92 g/m^2^ and presence of the cellulose component). The other side of the sensor block which remains exposed towards the environment is covered by a black paper to protect from any contamination and to avoid interference with the photodetector measurements (Figure 2a).

### 2.4. Characterization

Steady state absorption was recorded using Shimadzu UV2600 spectrophotometer. Field emission SEM (QUANTA FEG 250) was used to investigate the surface morphology of the prepared sensor. All the sensing experiments were repeated using the newly developed prototype MEGH.

### 2.5. Computational Study

The hydrophobic and hydrophilic soil matrices were simulated using the porous media solute transport module of the COMSOL Multiphysics simulator. An isotropic soil matrix with dimension of 10 m × 5 m was considered for the study in both cases. The porosity of both the hydrophobic and hydrophilic soil matrices was considered to be 0.4 [22]. The density of the clay type hydrophilic soil was considered to be 1400 kg/m^3^ [23] and the density of the sandy hydrophobic soil was considered as 1930 kg/m^3^ [23]. The adsorption coefficient of the hydrophilic system was considered to be 10 times that of the hydrophobic system [24]. PQ defines an inlet of length 1 m (see later) for the water flow within the soil matrix at a velocity of 4.427 m/s. Considering the rule of energy conservation, it was found that velocity of water flow through the inlet was 4.427 m/s (considering acceleration due to gravity to be 9.8 m/s^2^).

### 2.6. Development of an Intuitive Algorithm for Data Acquisition

The proposed device MEGH is preloaded with an indigenously developed Arduino-based interface for data acquisition and analysis (Figure 2b). The on-board microcontroller acquires data from the photodetector after turning on the R, G, B LEDs individually. The intensity of LED is based on the optical power source. The spectroscopic information is obtained in the form of amount of light absorbed in the red region, green region, and blue region. The acquired data were analysed by an intuitive algorithm which converts the spectroscopic information into the relative humidity based on the calibration equation fed to the software. The software is entirely developed in the Arduino platform for automated data acquisition and output result generation. After being powered up, health check-up and initialization of the instrument takes place. If there is any discrepancy, the device auto-corrects different conditions and restarts automatically. Next, the device turns on the RGB light individually and acquires the optical parameters from the developed sensor strip embedded inside the MEGH, placed at a distance of 20 cm from the soil. The device calculates the relative humidity of the soil, which is in equilibrium with the soil moisture. The device also acquires the temperature, humidity of the air, and soil moisture and stores the data in a library. The self-developed, IoT-enabled software uploads all data to the cloud for future big-data analysis and thus makes them available for prompt intervention, if required.

To implement an artificial intelligence (AI) framework for the developed sensing technique, a sophisticated machine learning (ML) protocol known as Artificial Neural Networks (ANN) was utilized. The input layers and output layers of the network are connected by single or multiple hidden layers (Figure 2c). In the input layer, the relatively dry, blue FMM are used, along with temperature sensor. The intermediate hidden layer (HL) 1 collects the RH information of the matrix by photodetection and by observing the colour change in FMM. It also compares the relative humidity acquired by the conventional system with that calculated by the FMM. If both the relative humidity levels are comparable, it moves to the next hidden layer (HL2). This ensures a double check on the estimation of the relative humidity and the need for intervention. HL2 stores crop-specific water requirement data. HL3 calculates the water potential from the reports generated by HL1. HL4, the decision-making layer, decides whether further hydration is required. Finally, the output layer is ready for intervention.

### 2.7. Caluculation of Accuracy, Limit of Detection (LOD) and Limit of Quantification (LOQ)

The accuracy, LOD, and LOQ of the device were calculated using the following Equation [25].

Accuracy = 100% − Error rate(2)

where error rate = (Observed Value − Actual Value)/Actual Value.

LOD = (3.3 × S.D.)/S(3)

LOQ = (10 × S.D.)/S(4)

where S.D. is the standard deviation and S is the slope of the calibration curve (Figure S1 in Supplementary Materials).

The precision of the instrument is expressed in terms of the standard deviation.

### 2.8. Statistical Analysis

All quantitative data are expressed as Mean ± Standard Deviation (SD) unless otherwise stated. GraphPad Prism v8.0 (GraphPad Software) and Sigmaplot v14.0 (Systat Software, Inc, Chicago, IL, USA) were used for statistical analysis. For all comparisons, a *p* value < 0.05 was considered statistically significant.

## 3. Results and Discussion

### 3.1. Characterization of the Nano Sensor

**Figure 3:**
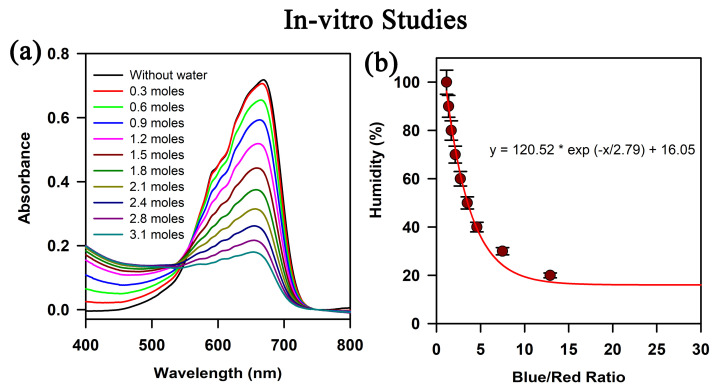
Interaction of cobalt chloride with water nano clusters: (**a**) Absorbance spectra of CoCl_2_ in DX and with increasing mole fraction of water. (**b**) Characteristic curve of interaction of the FMM with change in humidity in a closed chamber.

The absorption spectra of cobalt chloride (CoCl_2_) in pure dioxane (DX) and DX–water mixture are shown in Figure 3a. DX–water mixtures provide a suitable environment to monitor the dynamical properties of water molecules by modifying the dioxane-to-water ratio in the mixture. Although DX is a non-polar solvent, it cannot self-associate to form clusters. Instead, it is miscible with water by forming hydrogen bonds. On the other hand, formation of water nanoclusters in DX-water mixtures has been confirmed by previous studies by our group [26]. As the water content increases, the size of the water cluster grows and the local concentration of water molecules increases. The absorption spectra of CoCl_2_ vary with the change in relative concentration of water and DX solvent mixture, which suggests the dependence of CoCl_2_ on the polarity of the solvent. In pure DX solvent, there is a single absorbance peak at 650 nm, confirming the tetrahedral complex of cobalt ions ([CoCl_4_]^2−^) [27]. With the addition of water, the intensity of the band decreases and a new band appears at ~450 nm, which is a signature of octahedral complex species ([Co(H_2_O)_6_]^2+^) [27,28]. According to the quasi-lattice concept of salt in aqueous solution, the water molecules occupy the anionic sub lattice positions, resulting in a change in the tetrahedral geometry of Co^2+^ ions to octahedral geometry [28]. Thus, with increasing water content, the Co^2+^ ions experience a more polar environment in the form of water nanoclusters within DX–water mixtures, which finally changes its coordination geometry. The prepared sensor strips were subjected in a closed controlled humidity chamber and the corresponding colour change was quantified using a photodetector. It has been observed that the change in the relative ratio between the Co^2+^ ions in the tetrahedral geometry with that of the Co^2+^ ions in the octahedral geometry does not follow a linear relation with the mole fraction of water (Figure 3b). The non-linearity might arise from the non-linear dependence of the dielectric constant of the mixture on the mole fraction of water [29].

**Figure 4:**
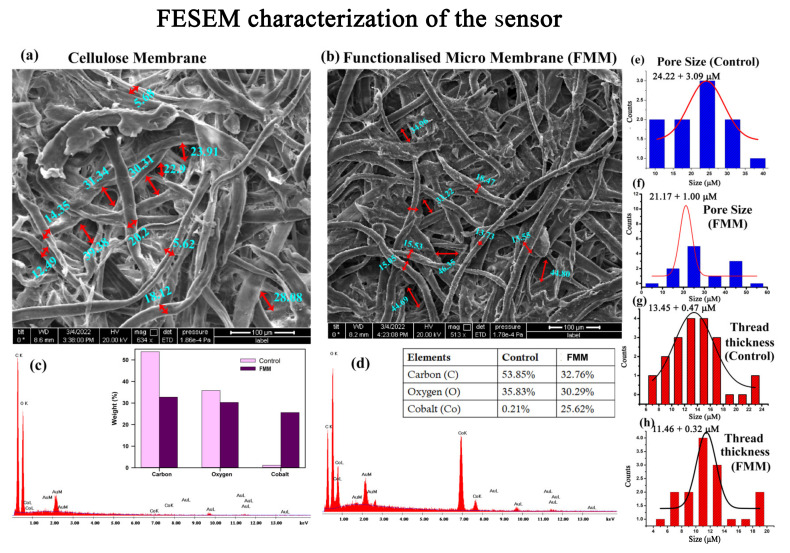
FESEM characterization of the FMM: (**a**,**b**) SEM micrographs of the cellulose fibre before and after functionalisation with CoCl_2_. (**c**,**d**) EDAX image of the selected FESEM area before and after functionalisation with CoCl_2_. Inset: The increase in weight percentage of the Co after functionalisation. (**e**,**f**) Pore size of the cellulose matrix before and after functionalisation with CoCl_2_. (**g**,**h**) Thread thickness of the cellulose matrix before and after functionalisation with CoCl_2_.

To understand the structural details of the prepared sensor, we performed field emission scanning electron microscopy (FESEM). Figure 4 shows the morphology of the fibres of the cellulose paper before and after functionalisation with CoCl_2_. The paper substrate was largely made of microscale cellulose fibrous of thickness 13.45 ± 0.47 µm strands interwoven with each other, making a pore size of 24.22 ± 3.09 µm (Figure 4e,g). After functionalisation, although the overall contrast of the cellulose matrix increased (Figure 4b), the pore size (11.46 + 0.32) and the thread thickness (21.17 + 1.00) did not show any significant change (Figure 4f,h). The energy dispersive X-ray (EDAX) analysis of the selected area in FESEM shows an incorporation of 25.62% cobalt within the cellulose matrix (Figure 4d inset) in comparison to the control. Thus, no significant change in the morphology of the cellulose matrix was observed after functionalisation with CoCl_2_. The porous structure and the hydrophilicity of the cellulose membrane facilitates the absorption and diffusion of the water molecules. This increases the chances of interaction of the cobalt ions with the water molecules [30].

### 3.2. Determination of Matric Potential and Simulation Studies

**Figure 5:**
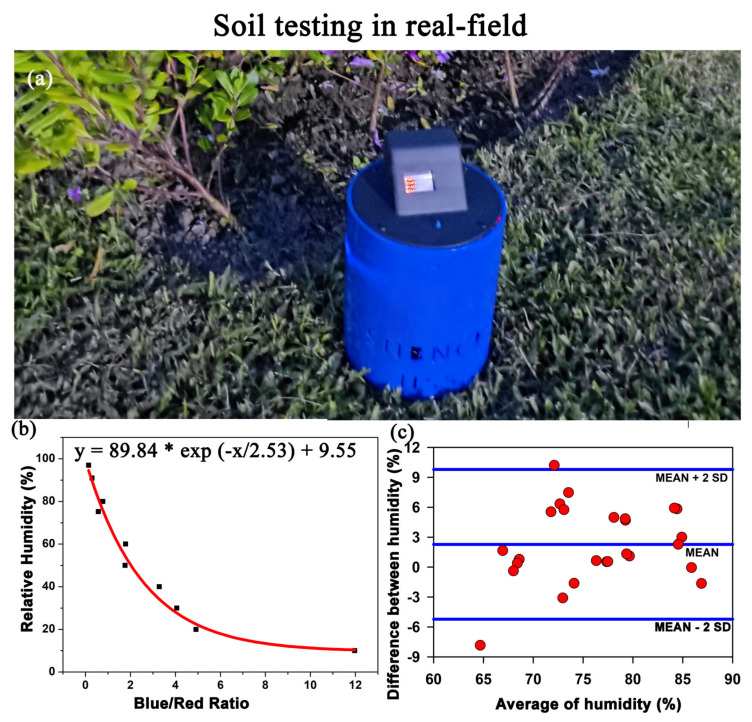
The development of the prototype “MEGH”: (**a**) The indigenously developed prototype “MEGH” for the estimation of soil moisture in a non-invasive manner. (**b**) Calibration curve of the device in response to various humidity condition in a closed chamber. (**c**) Bland–Altman analysis to estimate the standard deviation between the value obtained by the device and the conventional system.

We developed a device called “MEGH” to indirectly determine the moisture from a matrix (soil/cotton sample) by evaluating the relative humidity of the matrix (Figure 5a). Figure 5b shows the calibration of the device to evaluate the relative humidity of soil. With increase in moisture level, the colour of the developed sensor strip changes from blue to pink. The ratio of absorbance at 650 nm and 450 nm was taken as the instrumentation index and the calibration curve was obtained. Figure 5c shows the Bland–Altman analysis of the soil moisture values obtained from our developed device and the conventional soil moisture sensor. The mean ±2 SD values ranges from −4 to +9.5, which means a maximum SD of 9.5 units.

**Figure 6:**
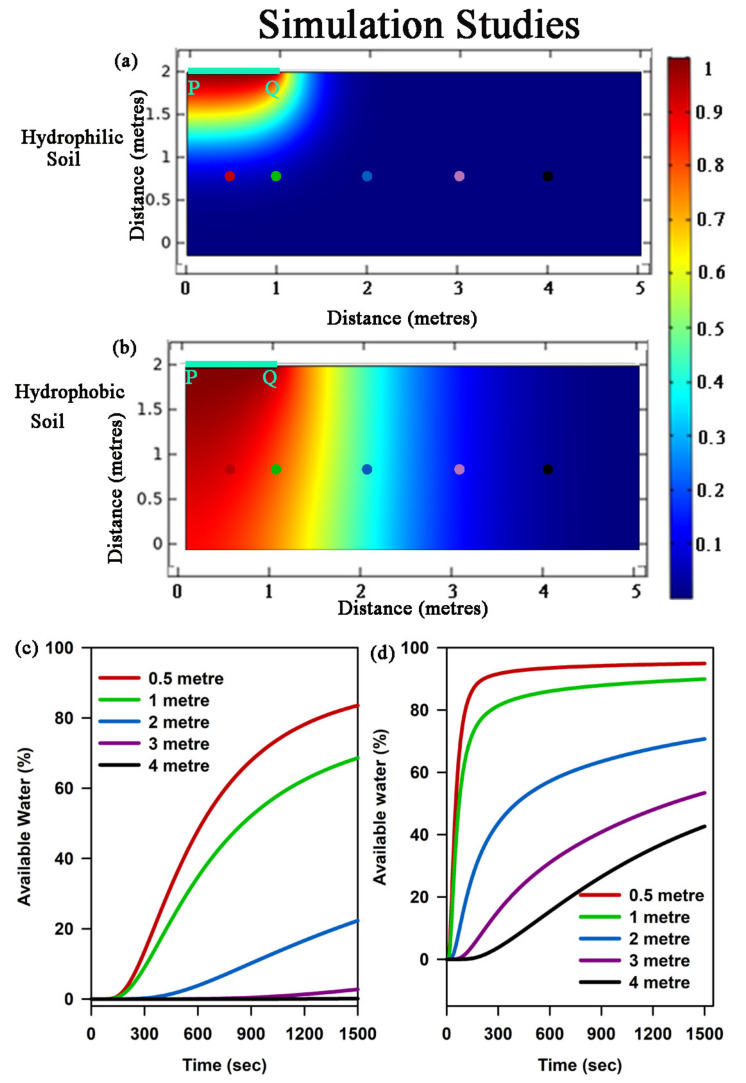
Simulation studies to evaluate the percentage of water availability in various soils: (**a**) and (**b**) Concentration gradient of water flow in a hydrophilic and hydrophobic soil matrix, respectively. PQ refers to the inlet for water flow within the soil matrix. The coloured dots signify the distance (in meters) within the soil matrix, where the flow of water has been observed in a time-dependent manner. (**c**) and (**d**) Characteristic curve of available water till 1500 s in both hydrophilic and hydrophobic soil matrix, respectively.

To study how the water disperses into the matrix and how much water is available to plants, we performed simulation studies in carefully chosen matrix environments. Figure 6 shows the movement of water in hydrophilic soil (Figure 6a) and hydrophobic soil (Figure 6b), where PQ is the inlet of the matrix environment in both the cases. After 300 s, it was observed that for hydrophilic soil, the water was locally confined, whereas for hydrophobic soil after the same time interval, the water was distributed substantially both along horizontal and vertical directions. This result signifies that in case of hydrophilic soil, due to the strong cohesion–adhesion forces between the water and the materials of the matrix, the moisture becomes confined in the neighbourhood of the inlet (Figure 6a). On the other hand, for a hydrophobic surface, due to the low interactive force, the water can spread to a higher extent (Figure 6b). The same fact becomes evident from Figure 6c,d, where available water percentage varies largely at different distances, with respect to time and with the change in matrix type. For example, at a distance of 4 m, available water percentage after 1500 s is less than 5% for a hydrophilic matrix, whereas for a hydrophobic matrix, the value is greater than 30%, suggestive of more plant-available water in hydrophobic soil.

**Figure 7:**
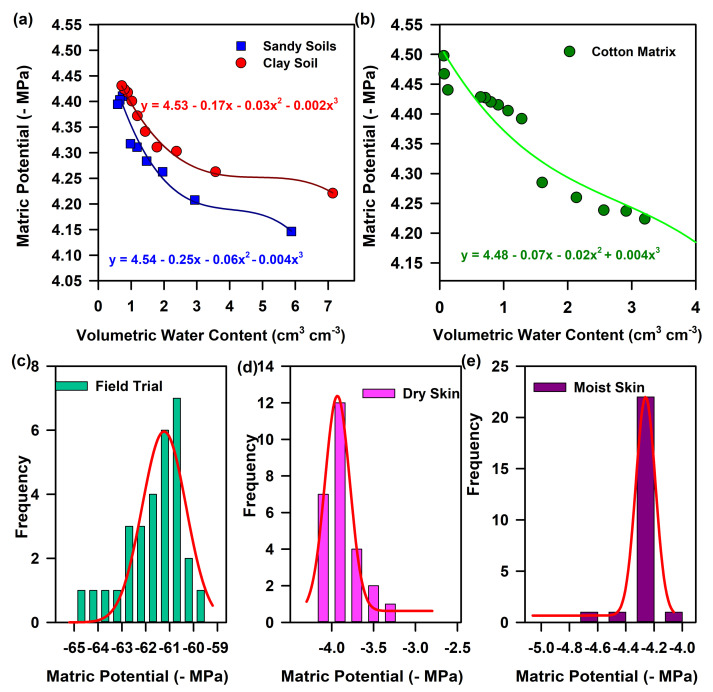
Assessment of water potential in various matrices: (**a**) Characteristic matric potential curve of sandy (hydrophobic) and loamy soil (hydrophilic). (**b**) Characteristic matric potential curve of cotton. (**c**) Evaluation of soil matric potential from 22 places in the same geographical area. (**d**) Determination of water potential in dry skin from 25 human subjects. (**e**) Determination of water potential in moist skin from 25 human subjects.

Using our developed device “MEGH”, we estimated the relative humidity of various soil environments and calculated the matrix potential using the Equation (1). Figure 7a,b shows the change in the matrix potential of two types of soil, namely, hydrophobic (sandy soil) and hydrophilic soil (clay soil). The plant-available water is the difference between the water content at field capacity, and the water content at the permanent wilting point, i.e., after drainage [1]. In the case of sandy soil, the amount of water available to the plants is much more as compared to clayey soil (Figure 7a). This can be due to the retention of water molecules on the surface of the soil grains due to adhesion and cohesion forces, which is much higher in hydrophilic soil (clay) than hydrophobic soil (sand). In order to validate the device, we evaluated the matric potential of different soil conditions using our device (Figure 7b). As the device was validated in a particular geographical area, the matric potential was found to be within −62 MPa to −65 MPa. The frequency distribution of the water potential from 22 places reveals a maximum occurrence of −61.2 MPa of water potential in the particular geographical area.

To establish the smallest amount of the analyte that can be efficiently measured by the device, the LOD, LOQ, precision, and intermediate precision were measured. The LOD and LOQ of the device were found to be −3.87 MPa and −12.27 MPa, respectively. This essentially means that although the device is able to quantify a minimum value of −3.87 MPa (LOD), −12.27 MPa (LOQ) is the minimum quantification limit of the device with an acceptable repeatability and trueness [31,32]. The precision or the standard deviation of the instrument was found to be mean ±1.14 MPa and the intermediate precision was found to be 1.84 MPa. This means that the instrument quantifies the water potential with a maximum fluctuation of ±1.14 units. The specificity of the instrument was determined on the basis of requirement of irrigation and was found to have a specificity of 95%. The accuracy of the device was calculated following Equation (2) and was found to be 72.15%. According to the validation of the device, the linearity range of the device to quantify water potential from soil was found to be between −60 to −64 MPa, based on a specific geographical area.

To establish a multipurpose application of the sensor strip as mentioned earlier, the performance of our developed FMM sensor as a biomedical aid was also investigated. For our study, cotton was used as a matrix, which maintains the timed delivery of hydration required by some biomedical devices. The matric potential of cotton was calculated by using Equation (1) to determine the amount of available water and retention capacity of cotton. We validated the device in 50 human subjects with both dry and moist skin. The matrix potential in dry skin was found to be less than −4 MPa, whereas in moist skin the matrix potential increased to −4.8 MPa indicative of moisture retention within the skin pores. The frequency distribution revealed a water potential of −3.9 MPa for dry skin and a water potential of −4.2 MPa for normal skin. A decreased water potential value in normal skin shows the retention of water on the skin surface. However, a higher water potential value signifies the lack of moisture adsorption on the skin surface for dehydrated or dry skin.

## 4. Conclusions

In summary: Fibrous cellulose membranes was functionalised successfully by using cobalt chloride, which serves as the colorimetric sensor for humidity. The CoCl_2_ concentration was found to have no influence on the morphology of the cellulose matrix. The membranes exhibit different colours in different humidity conditions, which was quantified by our developed prototype “MEGH”. The relation between the relative humidity above the soil surface and the soil moisture was established by measuring the humidity above both hydrophilic and hydrophobic soils using our developed device. The percentage of water availability for plants is greater in hydrophobic soil than in hydrophilic soil, which was also confirmed using simulation studies. In addition, the need for hydration for human skin was also evaluated by measuring the water potential of both dry and moist skin types. The developed device along with the prepared FMM sensor is believed to have dual application in the agricultural sector for measuring the water availability in different soil types and in the biomedical sector for measuring the timed requirement of application of hydration.

## Figures and Tables

**Table 1:** Comparative list of available instruments for measurement of water potential.

Type of Instrument	Working Principle	Components	Reference
Piezometers	Piezometric pressure calculation using Bernoulli’s law	Metal or plastic pipes inserted in soil matrix by drilling	[4]
Tensiometers	Young–Laplace Equation, which correlates between pore radius and water potential	Porous ceramic cup in contact with soil is installed at the end of a water-filled tube, and depending on the soil moisture, measurement of the water pressure is performed by a mechanical or electrical pressure sensor	[5]
Heat dissipation sensors	Thermal conductivity	Porous ceramic cup placed in contact with soil, resistor-like heat dissipating element, a temperature sensor	[6]
Granular matrix sensor	Electrical conductivity	Stainless steel sleeve, synthetic membrane, electrodes, handheld meter to measure conductivity	[7]
Dielectric sensors	Dielectric permittivity	Ceramic cup with pores, dielectric constant measurement kit	[8]
Thermocouple psychrometry	Kelvin Equation	Sealed chamber, metal thermocouple	[9]

## Data Availability

Data will be available from the corresponding author on request.

## Supplementary Materials

The following supporting information can be downloaded at: https://www.mdpi.com/article/10.3390/bios13020185/s1, Figure S1: Validation curve for quantification of water potential using the conventional system and the FMM sensor.
