# Supplementary material for: Sensing Bioavailable Water Content of Granulated Matrices: A Combined Experimental and Computational Study
Source: Biosensors (Basel). 2023 Jan 25;13(2):185. doi: 10.3390/bios13020185 (PMC9953787; doi:10.3390/bios13020185)
Supplement: Supplementary file 1 [file biosensors-13-00185-s001.zip › biosensors-2097300-supplementary.pdf]

Sensing Bioavailable Water Content of Granulated Matrices: A Combined Experimental and Computational Study

Ria Ghosh [1], Neha Bhattacharyya [2], Amrita Banerjee [3], Lopamudra Roy [4], Debdatta Mukherjee [5], Soumendra Singh [1], Arpita Chattopadhyay [6], Tapan Adhikari [7,*] and Samir Kumar Pal [1,*]

[1] Department of Chemical, Biological and Macromolecular Sciences, S. N. Bose National Centre for Basic Sciences, Kolkata 700106, India
[2] Department of Radio Physics and Electronics, University of Calcutta, Kolkata 700009, India
[3] Department of Physics, Jadavpur University, Kolkata 700032, India
[4] Department of Applied Optics and Photonics, University of Calcutta, Kolkata 700009, India
[5] Department of Biochemistry and Biophysics, University of Kalyani, Kalyani 741235, India
[6] Department of Basic Science and Humanities, Techno International, Kolkata 700156, India
[7] Indian Institute of Soil Science Nabibagh, Bhopal 462038, India
* Correspondence: tapan_12000@rediffmail.com (T.A.); skpal@bose.res.in (S.K.P.)

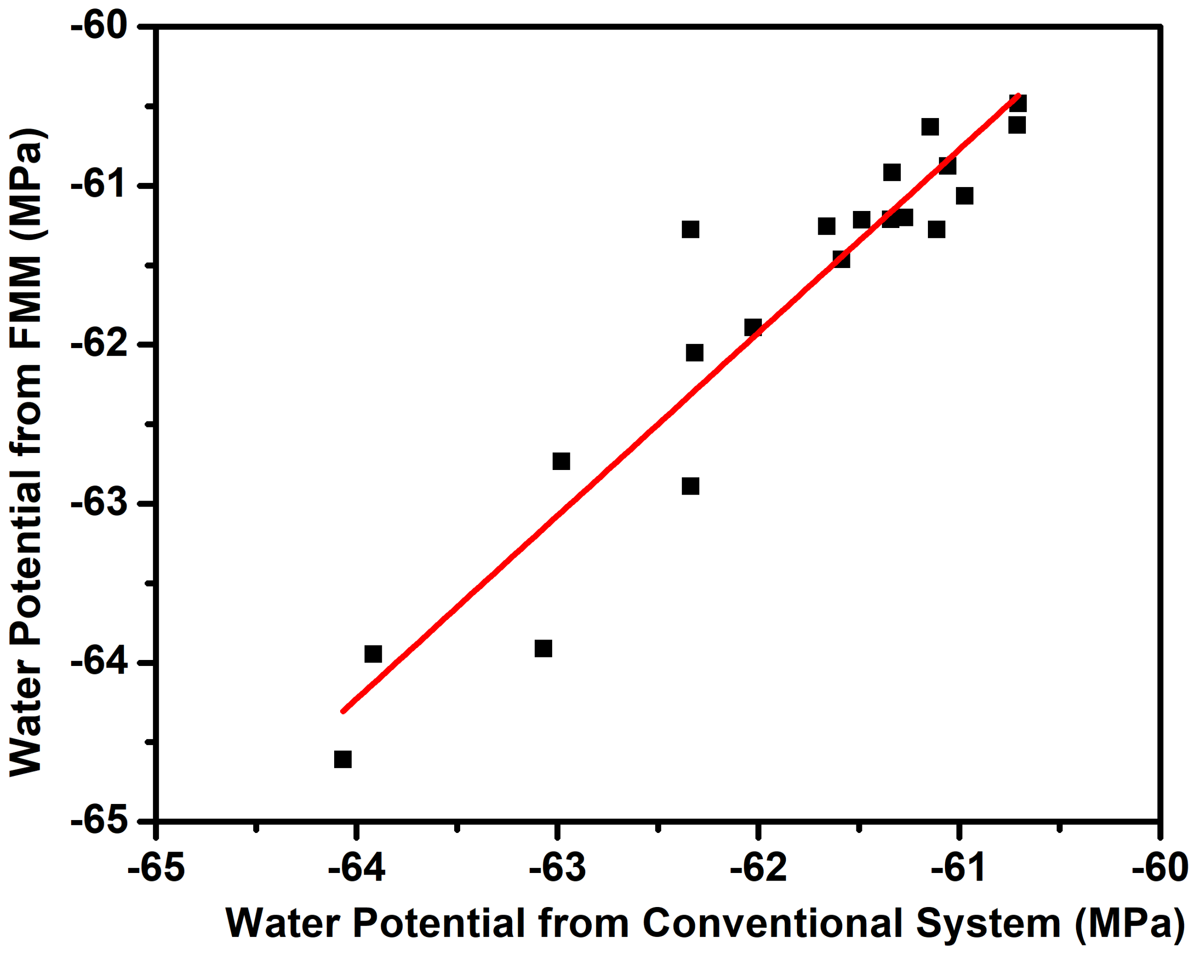

Figure S1: Validation curve for quantification of water potential using the conventional system and the FMM sensor.
